# Does students’ awareness of school-track-related stereotypes exacerbate inequalities in education?

**DOI:** 10.1038/s41539-023-00203-9

**Published:** 2023-12-14

**Authors:** Lisa Bardach, Claudia Neuendorf, Kou Murayama, Thorsten Fahrbach, Michel Knigge, Benjamin Nagengast, Ulrich Trautwein

**Affiliations:** 1 University of Tübingen, Hector Research Institute of Education Sciences and Psychology, Tübingen, Germany; 2 University of Potsdam, Education Department, Potsdam, Germany; 3 Humboldt University Berlin, Department of Rehabilitation Sciences, Berlin, Germany; 4 Korea University, Department of Education and the Brain & Motivation Research Institute (bMRI), Seoul, Republic of Korea

**Keywords:** Education, Human behaviour, Society

## Abstract

Early ability tracking increases inequalities in education. It has been proposed that the awareness of negative school-track-related stereotypes contributes to educational inequalities, as stereotype awareness interferes with students’ abilities to thrive, particularly those in lower, stigmatized tracks. The present study tested this assumption in a sample of 3880 German secondary school students from three tracks, who were assessed four times on stereotype awareness regarding their own school track and academic outcomes (achievement, engagement, self-concept) between Grades 5 and 8. Students in the lowest track reported higher levels of stereotype awareness than higher track students or students attending a combined track. Stereotype awareness increased across time in all tracks. Contrary to our preregistered hypotheses, however, the results from multigroup models revealed that (changes in) stereotype awareness were not more strongly related to (changes in) most outcomes in the lowest track in comparison with the other two tracks.

## Introduction

Many education systems all around the globe group students by ability. Often, the sorting of students into different educational tracks is viewed as a way to help educators target students’ learning needs more effectively. At the same time, tracking has lasting consequences for students’ learning and later careers. For instance, educational tracks represent differential developmental contexts, with higher average levels of teaching quality and learning rates in higher tracks^1,2^. Further, the assignment to different school tracks has been criticized for reproducing existing social class differences^2,3^, as children from less socioeconomically advantaged families have a higher chance of being enrolled in a lower track secondary school. These tracking decisions cannot be explained by lower abilities of disadvantaged groups alone, as it has been shown that teachers provide higher tracking recommendations for students coming from higher socioeconomic status backgrounds than for equally performing students with lower socioeconomic status^4^.

In addition, students’ awareness of stereotypes relating to their school track could further exacerbate inequalities. Stereotypes capture oversimplified beliefs about the characteristics of members of certain groups^5^, and educational tracks generate different stereotypes about the students attending these tracks. Stereotypes regarding students in higher ability tracks (higher status) involve the characterizations that they are “smart” and “perform well,” whereas the opposite is expected from students attending lower ability tracks (lower status) for whom negative stereotypes prevail (e.g., being “stupid or “unmotivated”^3,5–7^). Being aware of negative stereotypes can lead to disengagement from the stigmatized domain and lower domain-specific performance^7,8^. Hence, awareness of negative school-track-related stereotypes may become a psychological barrier that particularly hinders the thriving of lower track students.

However, prior quantitative research on students’ awareness of school-track-related stereotypes has been surprisingly scarce^3,7,9^, leaving serious gaps in the current understanding. The present longitudinal study therefore set out to investigate (a) mean-level differences in students’ awareness of negative school-track-related stereotypes (comprising negative cognitive, motivational, and social stereotypes) between tracks, (b) developmental trajectories of stereotype awareness, and (c) relationships between (changes in) stereotype awareness and (changes in) academic outcomes in terms of academic achievement (standardized achievement test scores), self-concept of academic aptitude, and school engagement. We relied on a sample of German secondary school students from three different nonacademic tracks who were assessed four times on their awareness of stereotypes and all outcomes between the ages of 11 and 14 years (Grades 5, 6, 7, and 8). Specifically, our sample included students from the German federal state of Baden-Württemberg attending Hauptschule, the lowest track in Germany, and Realschule, a higher track. Our sample also included students from Mittelschule, a combined track (students in this track could get a diploma equivalent to Hauptschule or a diploma equivalent to Realschule) from the German federal state of Saxony. Multigroup models were used to examine potentially differentiated patterns of effects in different school tracks. Throughout the manuscript, students’ awareness of school-track-related stereotypes refers to stereotypes pertaining to a student’s own school track (i.e., students from a particular track report how aware they are of stereotypes that pertain only to their track) and not stereotypes that pertain to other tracks.

Does students’ awareness of school-track-related stereotypes differ between tracks (*Research Question 1, RQ1*)? The placement in a relatively lower track (compared with a relatively higher track) can be perceived as a devalued social position reflective of a student’s ability and includes information about the student’s standing in society^10–12^. Tracking thus provides students with institutionalized status labels that are highly visible and impactful (e.g., with respect to later career chances and life paths^9,13^). Moreover, students know about the image of their track in society. Consequently, lower track students are aware of the negative stereotypes that “others” or “people in general” hold about their school track^7,9^, even though they do not necessarily endorse these negative stereotypes themselves (see also research on social stigma^14^).

Differences in stereotype awareness between tracks, with higher levels of stereotype awareness in lower tracks, as well as the content of stereotypes can also be linked to differentiation-polarization theory^10,15,16^. The theory states that the differentiation of students into different tracks leads to a polarization of the students’ school attitudes. For higher track students, school is a positive experience, given that belonging to a higher track reflects a higher status. By contrast, lower track students lose status due to their assignment to a lower and less valued track. Lower track students therefore react against this system and the values it upholds, namely, ability and hard work. Consequently, an “antischool culture” emerges in the lower tracks^10,12,15^. Here, we argue that polarization might be reflected not only in school cultures and the corresponding school attitudes (as in the initial formulation of the theory) but also in students’ awareness of how the public perceives their track and the characteristics of students from their track, with negative characteristics that are detrimental to academic success (akin to antischool cultures and attitudes) being attributed to lower track students. Hence, regarding the preregistered RQ1 (differences between tracks), we hypothesized that we would find mean differences in stereotype awareness between tracks in all four waves, with higher average levels of negative school-track-related stereotype awareness for students from the lowest track in comparison with students from the combined track and the higher track.

How does students’ awareness of school-track-related stereotypes develop over time in different tracks (*Research Question 2, RQ2*)? Conceptually, stereotypes have often been considered to be fixed, persisting even in the face of conflicting evidence^17^. Thus, students’ awareness of school-track-related stereotypes could remain stable over time, reflecting stable status differences between tracks within a society that are visible to students. On the other hand, it has been highlighted that stereotypes can undergo developmental changes during the school years^18,19^. For students’ awareness of school-track-related stereotypes, both increasing and decreasing trajectories seem theoretically plausible.

Students’ awareness of negative school-track-related stereotypes may increase over time, at least in the lower tracks. During adolescence, students gain a more nuanced understanding of their own social position and become more sensitive to cues reflecting the devalued position of their group (here: lower track students) in society^5,20^. These developmental processes likely provide a fertile foundation for lower track students’ stereotype awareness. Alternatively, stereotype awareness could weaken across the secondary school years, even in the lower tracks. As soon as students are placed in a track at the beginning of secondary school, their salient reference group shifts over time from the entire age cohort to only those students in one’s own track^21^. The reality of students’ daily lives and interactions with diverse others in their track could alleviate initial negative beliefs about the image of their track, manifesting in decreasing trajectories for stereotype awareness^22^.

However, considering the current lack of longitudinal research that has explored how students’ awareness of school-track-related stereotypes in different school tracks develops across several years and the contrasting theoretical assumptions about such trajectories, it is difficult to derive clear predictions. Therefore, for the preregistered RQ2 (changes in students’ awareness of school-track-related stereotypes over time in the three different tracks), we did not specify concrete hypotheses and conducted exploratory analyses instead.

Lastly, how is students’ awareness of school-track-related stereotypes related to academic outcomes (*Research Questions 3–5, RQ3-5*)? In the current study, we investigated three types of academic outcomes: Academic achievement in terms of standardized achievement test scores, students’ self-concept of academic aptitude, and school engagement. Below, we outline theoretical considerations and prior research findings on relationships between the awareness of school-track-related stereotypes and academic outcomes, with an emphasis on potential differences between tracks.

The awareness of school-track-related stereotypes may amplify inequalities, as stereotype awareness could be particularly harmful to members of stigmatized groups^23^. Due to the social stigma associated with their track, it is plausible that lower track students’ awareness of negative school-track-related stereotypes negatively affects their school-related development (academic achievement, engagement, self-concept). Potential mechanisms underlying these negative effects among lower track students are, for example, stereotype threat and self-fulfilling prophecies^8,24^ (but see refs. ^3,25^; for research that questioned the relevance of stereotype threat in “real-life” settings). On the other hand, given that higher track students belong to a nonstigmatized (higher status) track, they might not be affected by negative stereotypes that refer to the “typical” higher track student. To conclude, according to what we call the “stereotype awareness as an amplifier of inequality” hypothesis, the awareness of school-track-related stereotypes hampers the thriving of students from the lowest track. Hence, stereotype awareness should be most strongly and negatively related to the academic outcomes of students in the lowest track, thereby increasing the inequalities that are associated with the different school tracks.

Whereas the “stereotype awareness as an amplifier of inequality” hypothesis primarily builds on social psychological and sociological theories, an individual differences perspective supports a competing hypothesis. Such a competing hypothesis could state that group categories and objective status differences associated with different tracks may be less important, and instead, individuals’ subjective perceptions of negative school-track-related stereotypes matter most (see also, e.g., research on effects of subjective socioeconomic status^26,27^). This implies that school-track-related stereotype awareness might not be exclusively maladaptive for students from the lowest track. Rather, stereotype awareness may be negatively related to academic outcomes of individuals from other tracks, too, as long as they believe that such stereotypes exist; an assumption we call the “stereotype awareness as harmful for all” hypothesis.

Regarding links to academic achievement, a third assumption seems plausible. As students are sorted into different tracks on the basis of achievement, two scenarios can be outlined for achievement. Even though stereotype awareness may be negatively related to achievement, it is also reasonable to assume that relatively higher achieving students in a track are particularly likely to devalue their track and generalize this devaluing to how others perceive their track because, for them, the next higher track may also have been an option (from herein labeled the “stereotype awareness as an indicator of missed opportunities” hypothesis). In line with Big Fish Little Pond reasoning^28^ such a student could be the “big fish” (a relatively high-achieving student) in a “little pond” (their current track); however, as they did not make it to the “big pond” (next higher track), they devalue their current track and believe that other people in general do so as well.

Prior research on school-track-related stereotype awareness and academic outcomes able to test different theoretical assumptions has been limited. In a sample containing predominantly lower track students (Hauptschule, 72%) but not as many students attending the highest ability track in Germany (Gymnasium, 28%), awareness of school-track-related stereotypes was significantly and negatively correlated with students’ self-concept of academic aptitude, motivation, and academic achievement^7^. However, the cross-sectional design prevented conclusions from being drawn about developmental relationships between stereotype awareness and critical outcomes. In addition, school track was included as a predictor, but relationships between students’ awareness of school-track-related stereotypes and outcome variables were not explored separately for different school tracks^7^.

**Fig. 1:**
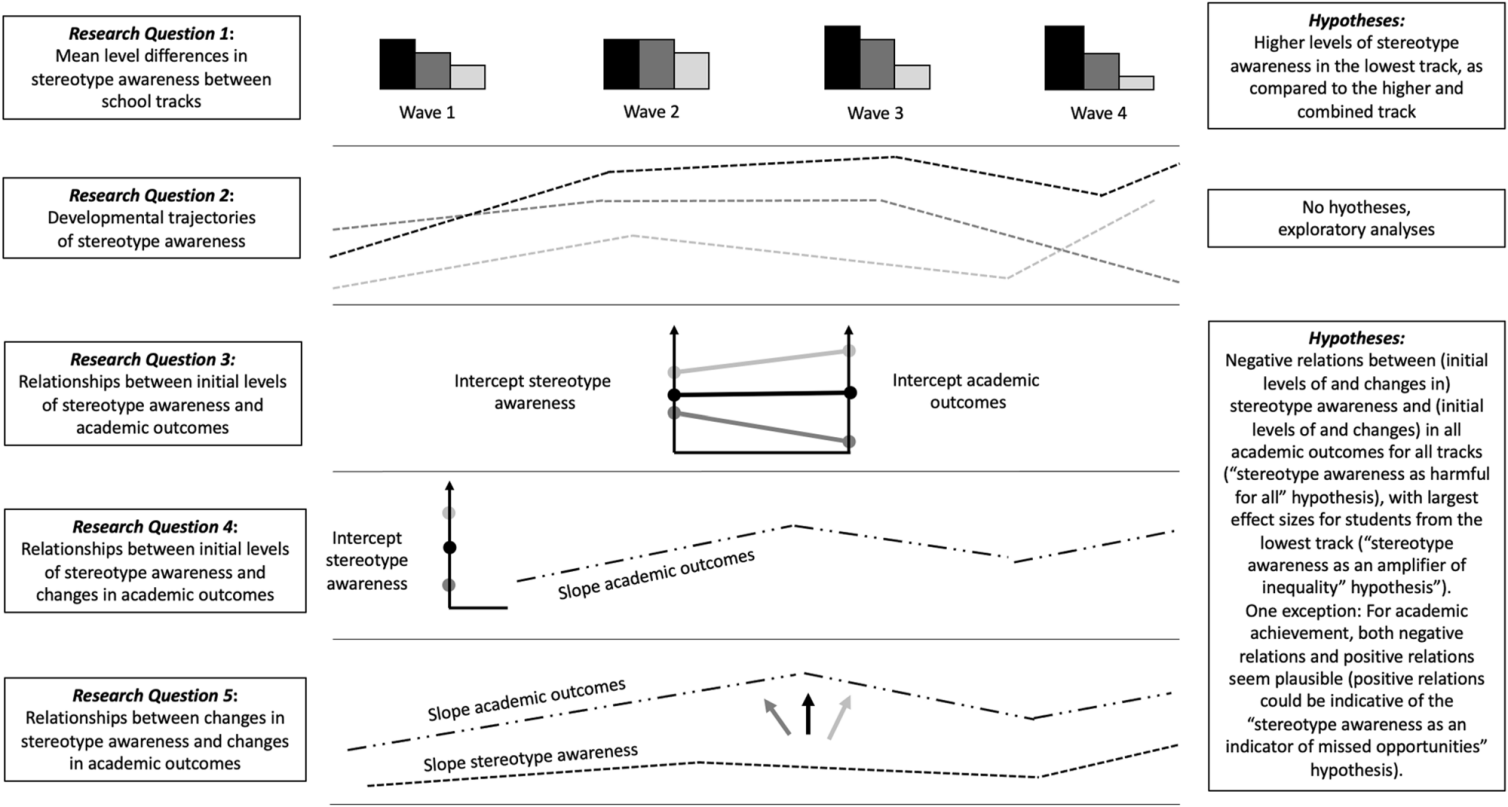
Research questions and hypotheses. Overview of the five research questions (left side) and hypotheses (right side) addressed in this study.

In light of the scarce amount of research on relationships between school-track-related stereotype awareness and academic outcomes, it seemed most appropriate to rely on theoretical considerations (in terms of the above outlined three hypotheses) to guide the current study’s preregistered research questions. Hence, RQ3 (relationships between initial levels of stereotype awareness assessed in Grade 5 right after students transitioned to secondary school and initial levels of academic outcomes), RQ4 (relationships between initial levels of stereotype awareness and developmental trajectories in outcomes variables), and RQ5 (relationships between changes in stereotype awareness and changes in outcome variables), we combined the “stereotype awareness as an amplifier of inequality” hypothesis and the “stereotype awareness as harmful for all” hypothesis. Thus, we hypothesized that (initial levels of and changes in) stereotype awareness should be negatively related to (initial levels of and changes in) all academic outcomes; however, while we assumed that the effects would be present in all investigated tracks (“stereotype awareness as harmful for all” hypothesis), we proposed that effect sizes should be largest for students from the lowest track in line with the “stereotype awareness as an amplifier of inequality” hypothesis. We hypothesized that there would be negative relationships between school-track-related stereotypes and the outcomes, with the potential exception of achievement scores. Whereas we expected to find significant relationships with achievement, we left it open whether these would be negative or, reflecting the “stereotype awareness as an indicator of missed opportunities” hypothesis, positive. Figure 1 provides an overview of all five research questions. Except for RQ1, which was addressed with tests of mean differences in stereotypes between tracks, all research questions were addressed with multigroup growth curve analysis. For RQs 3–5, we controlled for effects of potentially confounding variables (socioeconomic status, gender, migration background). This study’s research questions, hypotheses, and main analyses were preregistered on the Open Science Framework prior to the main analyses (on December 7, 2022, https://osf.io/uwjrm/). All analysis codes can also be found on the OSF.

## Results

### Bivariate correlations and descriptive information

Bivariate correlations are reported separately for the three tracks in Supplementary Figs. 1–3 in the Online Supplement. The student composition in the three tracks showed that students from the lowest track had lower SES backgrounds (*M*_SES_ = 43.21, *SD* = 11.86, measured as the highest socio-economic index of occupational status of the parents, HISEI^29^, which integrates information on income and education and can range from 16 [cleaner] to 90 [judge]) and were more likely to come from a migration background (46.8%) than students in the other two tracks (higher track: *M*_SES_ = 49.46, *SD* = 13.44, 16.9% migration background; combined track: *M*_SES_ = 45.54, *SD* = 12.11, 4.6% migration background). The gender composition was very similar, with 56%, 55%, and 53% male students in the lowest, combined, and higher tracks, respectively. Descriptive statistics (*M* and *SD* of all variables) and information on missing data can be found in Supplementary Table 1. We also calculated intraclass correlation coefficients for the stereotype awareness measure separately for each track for the four waves to provide additional information. ICC(1) values ranged from 0.000 to 0.081. Overall, little variance could be attributed to the classroom level, indicating that school-track-related stereotypes are best viewed as an individual-student-level construct and not as a group-level (i.e., classroom-level) phenomenon. Measurement invariance over time and across tracks was tested and generally supported (for details, see the “Method” section).

We deviated from the preregistered main analyses in two significant ways: First, we had preregistered that we could use school belonging as an outcome; however, due to persistent model convergence problems, we decided not to include the results for school belonging in this paper. Second, the analyses were based on a one-factor model for stereotype awareness comprising cognitive, social, and motivational stereotypes and not on separate factors (see the “Method” section for more details on the CFAs for stereotype awareness).

### Mean differences in stereotype awareness between tracks (RQ1)

**Fig. 2:**
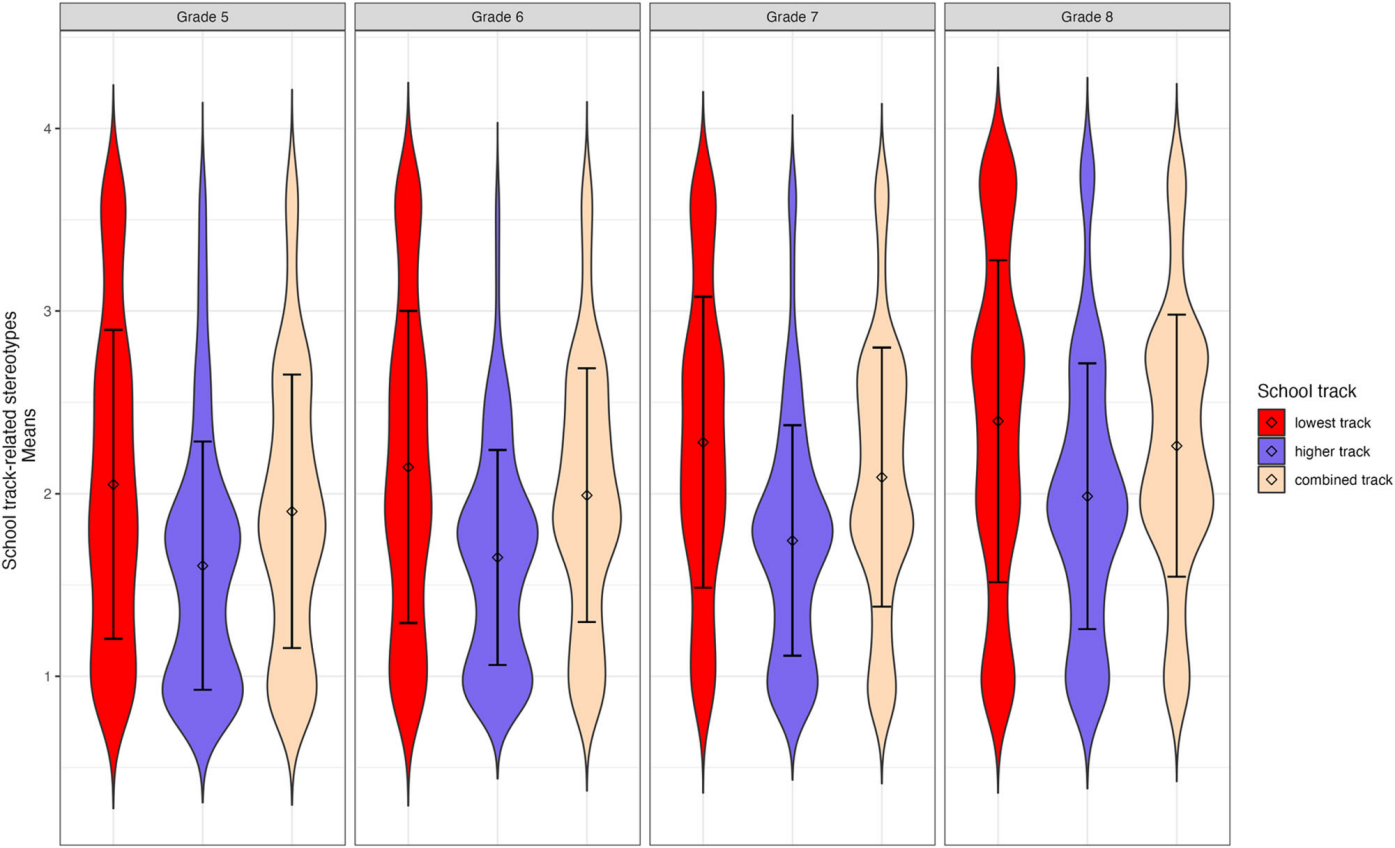
Mean-level differences. Violin plots displaying mean-level differences (including standard deviations) in school-track-related stereotype awareness between the three secondary school tracks for all four waves.

Tests based on model comparisons revealed significant mean differences in latent stereotype awareness factors between tracks for all waves (models with constrained means between tracks had a significantly worse fit than models with unconstrained means, χ^2^(2) ranging from 66.58 to 216.63, all *p*s < 0.001). Follow-up tests with a Benjamini-Hochberg correction to adjust for multiple testing indicated that students from the lowest track reported significantly higher levels of stereotype awareness in all waves than students from the higher and combined tracks (all *p*s < 0.001, except for the comparisons between the lowest and combined tracks in Grade 5: *p* = 0.002, Grade 6: *p* = 0.003, and Grade 8: *p* = 0.011). Students from the combined track further reported significantly higher levels of stereotype awareness than students from the higher track in all waves (all *p*s < 0.001). Figure 2 presents the mean-level differences.

### Developmental trajectories of stereotype awareness (RQ2)

**Fig. 3:**
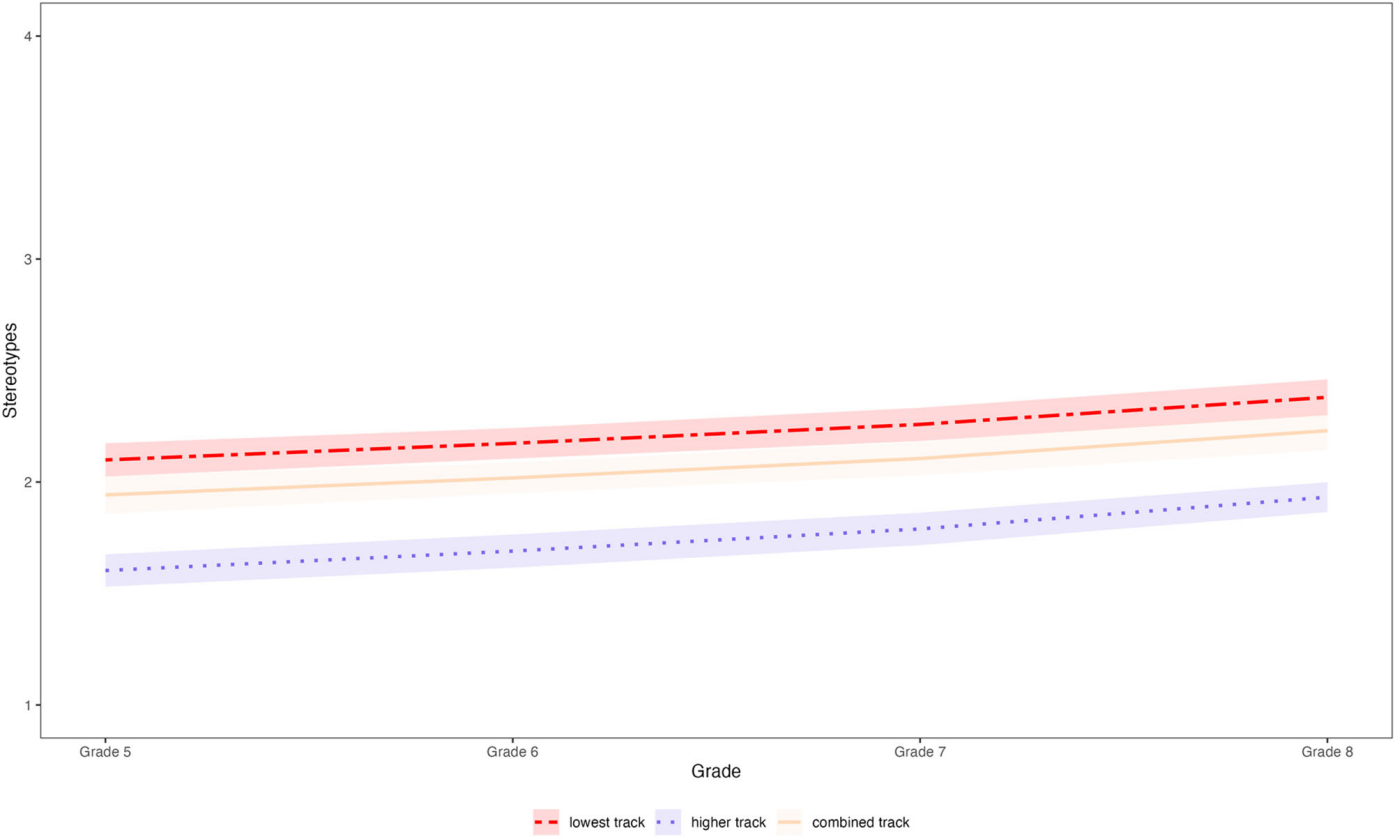
Developmental trajectories. The developmental trajectories (including 95% CIs) in school-track-related stereotype awareness from Grade 5 (first year of secondary school) to Grade 8 in the three tracks.

Multigroup univariate growth curve models were set up to investigate developmental trajectories in school-track-related stereotype awareness (see Supplementary Table 5 for details about growth curve model parameters). Stereotype awareness significantly increased in all tracks across the 4-year period (lowest track: *b* = 0.281; higher track: *b* = 0.329; combined track: *b* = 0.288; all *p*s < 0.001, see Fig. 3). To quantify the magnitude of the change, we additionally calculated Glass’s ∆ as an effect size indicator^30,31^. Glass’s ∆ amounted to 0.306, 0.447, and 0.371 for the lowest track, the higher track, and the combined track, respectively (all *p*s < 0.001). These findings indicate that the mean levels of stereotype awareness in all three tracks increased by roughly one third of a standard deviation across the 4 years. Moreover, comparing a model constrained to be equal across tracks with an unconstrained model revealed that the developmental trajectories did not differ significantly between tracks, χ^2^(2) = 0.912, *p* = 0.634.

### Relationships between initial levels of and changes in stereotype awareness and academic development (RQ3–RQ5)

**Fig. 4:**
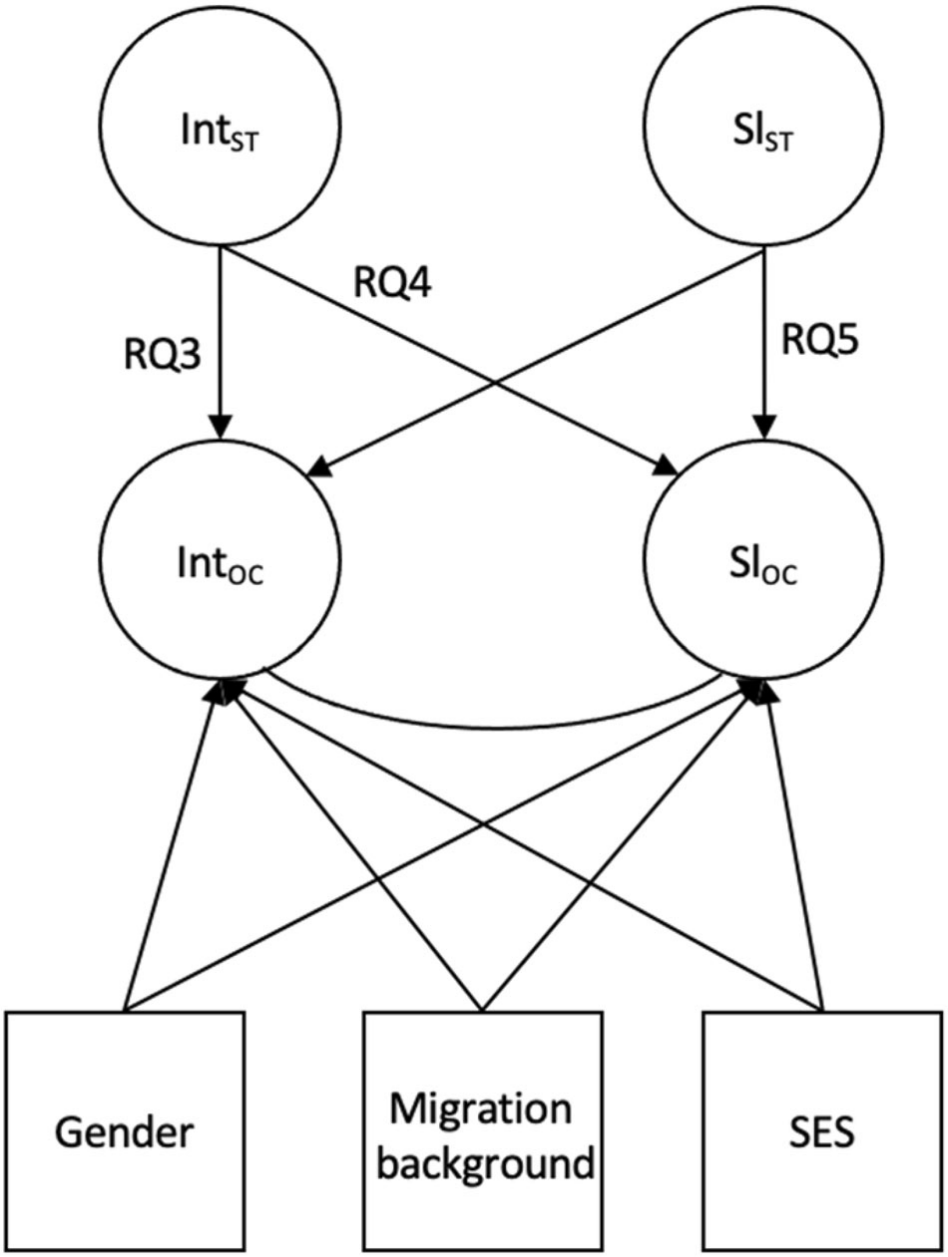
Multigroup latent growth curve model for investigating RQ3–RQ5. RQ3 addresses relationships between initial levels (intercept) of stereotype awareness and initial levels (intercept) of outcome variables in Grade 5, RQ4 addresses relationships between initial levels (intercept) of stereotype awareness in Grade 5 and changes (slope) in outcome variables, RQ5 addresses relationships between changes (slope) in stereotype awareness and changes (slope) in outcome variables. Int intercept stereotype awareness, Sl_ST_ slope stereotype awareness, Int_OC_ intercept outcome, Sl_OC_ slope outcome. The control variables gender, migration background, and socioeconomic status (SES) are displayed. Correlations between exogenous variables are not shown.

We estimated multigroup growth curve models to investigate the relationships between the initial levels of stereotype awareness and academic outcomes (RQ3), relationships between initial levels of stereotype awareness and changes in outcomes (RQ4), and relationships between changes in stereotype awareness and outcomes (RQ5), controlling for SES, gender, and migration background (Fig. 4). Separate models were set up for each outcome, and all three research questions were addressed in one model. The results are summarized in Table 1 (school engagement), Table 2 (academic achievement), and Table 3 (self-concept of academic aptitude).

**Table 1:** Multigroup growth curve model results for school engagement, with initial levels of stereotype awareness (intercept) predicting initial levels of engagement (intercept) for RQ3, initial levels of stereotype awareness (intercept) predicting changes (slope) in engagement for RQ4, and changes (slope) in stereotype awareness predicting changes (slope) in engagement for RQ5.

School track	Lowest track				Combined track				Higher track			
Parameter	*b*	SE	Est/SE	*p*	*b*	SE	Est/SE	*p*	*b*	SE	Est/SE	*p*
**Intercept of school engagement**												
**RQ3:** Intercept of stereotypes	**−0.360**	**0.092**	**−3.925**	**<0.001**	**−0.469**	**0.12**	**−3.909**	**<0.001**	**−0.594**	**0.100**	**−5.935**	**<0.001**
Slope of stereotypes	−0.051	0.239	−0.211	0.833	−0.154	0.144	−1.068	0.285	0.139	0.209	0.665	0.506
Gender	**−0.253**	**0.054**	**−4.661**	**<0.001**	**−0.181**	**0.064**	**−2.846**	**0.004**	−0.111	0.069	−1.610	0.108
Socioeconomic background	−0.024	0.036	−0.683	0.495	0.013	0.033	0.393	0.695	0.012	0.038	0.321	0.748
Migration background	**0.191**	**0.069**	**2.761**	**0.006**	−0.049	0.186	−0.263	0.793	**0.340**	**0.116**	**2.935**	**0.003**
**Changes in school engagement (slope)**												
**RQ4:** Intercept of stereotypes	−0.034	0.109	−0.309	0.833	−0.068	0.127	−0.535	0.833	0.083	0.128	0.647	0.833
**RQ5:** Slope of stereotypes	−0.005	0.278	−0.018	0.986	−0.011	0.155	−0.072	0.986	−0.288	0.215	−1.340	0.424
Gender	0.135	0.071	1.883	0.060	0.063	0.071	0.890	0.373	0.069	0.083	0.826	0.409
Socioeconomic background	0.010	0.043	0.228	0.819	−0.002	0.04	−0.057	0.955	0.013	0.039	0.325	0.745
Migration background	−0.147	0.08	−1.829	0.067	0.172	0.178	0.966	0.334	−0.173	0.096	−1.811	0.070

RQ = research question; Gender: 0 = female, 1 = male; Migration background: 0 = without, 1 = with; Statistically significant *p*-values (*p* < 0.05) are printed in bold. The Benjamini-Hochberg correction was applied to all *p*-values related to each research question, separately.

**Table 2:** Multigroup growth curve model results for academic achievement, with initial levels of stereotype awareness (intercept) predicting initial levels of achievement (intercept) for RQ3, initial levels of stereotype awareness (intercept) predicting changes (slope) in achievement for RQ4, and changes (slope) in stereotype awareness predicting changes (slope) in achievement for RQ5.

School track	Lowest track				Combined track				Higher track			
Parameter	*b*	SE	Est/SE	*p*	*b*	SE	Est/SE	*p*	*b*	SE	Est/SE	*p*
**Intercept of school achievement**												
**RQ3:** Intercept of stereotypes	**0.647**	**0.099**	**6.566**	**<0.001**	0.126	0.093	1.363	0.222	−0.090	0.108	−0.832	0.405
Slope of stereotypes	**0.469**	**0.185**	**2.539**	**0.011**	0.076	0.089	0.848	0.396	0.313	0.195	1.609	0.108
Gender	**0.173**	**0.050**	**3.496**	**<0.001**	**0.131**	**0.059**	**2.211**	**0.027**	**0.14**	**0.044**	**3.157**	**0.002**
Socioeconomic background	0.035	0.027	1.307	0.191	**0.088**	**0.027**	**3.227**	**0.001**	0.055	0.029	1.906	0.057
Migration background	**−0.268**	**0.076**	**−3.540**	**<0.001**	**−0.391**	**0.141**	**−2.781**	**0.005**	**−0.181**	**0.092**	**−1.967**	**0.049**
**Changes in school achievement (slope)**												
**RQ4:** Intercept of stereotypes	−0.17	0.109	−1.566	0.352	**−0.314**	**0.112**	**−2.799**	**0.046**	−0.289	0.133	−2.170	0.135
**RQ5:** Slope of stereotypes	−0.201	0.230	−0.875	0.572	0.164	0.127	1.290	0.424	−0.315	0.147	−2.144	0.288
Gender	**−0.249**	**0.064**	**−3.897**	**<0.001**	**−0.184**	**0.076**	**−2.439**	**0.015**	**−0.305**	**0.079**	**−3.868**	**<0.001**
Socioeconomic background	−0.015	0.037	−0.405	0.685	0.017	0.027	0.606	0.544	−0.022	0.041	−0.549	0.583
Migration background	**−0.213**	**0.087**	**−2.438**	**0.015**	0.047	0.215	0.219	0.827	0.015	0.107	0.136	0.892

RQ = Research Question. Gender: 0 = female, 1 = male; Migration background: 0 = without, 1 = with; Statistically significant *p*-values (*p* < 0.05) are printed in bold. The Benjamini-Hochberg correction was applied to all *p*-values related to a research question (separately for each research question).

**Table 3:** Multigroup growth curve model results for academic self-concept, with initial levels of stereotype awareness (intercept) predicting initial levels of self-concept (intercept) for RQ3, initial levels of stereotype awareness (intercept) predicting changes (slope) in self-concept for RQ4, and changes (slope) in stereotype awareness predicting changes (slope) in self-concept for RQ5.

School track	Lowest track				Combined track				Higher track			
Parameter	*b*	SE	Est/SE	*p*	*b*	SE	Est/SE	*p*	*b*	SE	Est/SE	*p*
**Intercept of academic self-concept**												
**RQ3:** Intercept of stereotypes	−0.069	0.068	−1.016	0.348	−0.137	0.074	−1.856	0.095	**−0.251**	**0.073**	**−3.444**	**0.001**
Slope of stereotypes	0.005	0.111	0.041	0.967	0.075	0.076	0.99	0.322	0.048	0.102	0.467	0.640
Gender	**0.112**	**0.034**	**3.309**	**0.001**	0.066	0.041	1.606	0.108	0.042	0.039	1.06	0.289
Socioeconomic background	0.024	0.020	1.198	0.231	0.009	0.019	0.482	0.630	0.028	0.019	1.478	0.140
Migration background	−0.027	0.044	−0.624	0.533	−0.040	0.130	−0.304	0.761	0.050	0.049	1.036	0.300
**Changes in academic self-concept (slope)**												
**RQ4:** Intercept of stereotypes	0.018	0.085	0.211	0.833	0.042	0.081	0.516	0.833	0.019	0.068	0.282	0.833
**RQ5:** Slope of stereotypes	−0.104	0.152	−0.683	0.636	−0.102	0.086	−1.186	0.424	−0.182	0.109	−1.674	0.424
Gender	−0.073	0.047	−1.533	0.125	0.030	0.042	0.715	0.474	**0.118**	**0.041**	**2.900**	**0.004**
Socioeconomic background	−0.020	0.024	−0.838	0.402	0.011	0.022	0.504	0.614	−0.035	0.025	−1.414	0.157
Migration background	0.041	0.053	0.777	0.437	0.185	0.145	1.282	0.200	−0.014	0.045	−0.309	0.757

RQ = research question. Gender: 0 = female, 1 = male; Migration background: 0 = without, 1 = with; Statistically significant *p*-values (*p* < 0.05) are printed in bold. The Benjamini-Hochberg correction was applied to all *p*-values related to a research question (separately for each research question).

#### Engagement

Initial levels of school-track-related stereotype awareness in Grade 5 were significantly and negatively related to initial levels of engagement in the lowest track *(b* = −0.360, *p* < 0.001), the higher track *(b* = −0.594, *p* < 0.001), and the combined track *(b* = −0.469, *p* < 0.001; RQ 3). However, initial levels of stereotypes were not significantly related to students’ development in engagement across the 4 years (RQ 4), and the developmental trajectories of stereotype awareness and engagement were not significantly related in any of the three tracks (RQ 5). Model comparisons revealed that a more parsimonious model in which parameters were constrained to be equal across tracks did not show a significantly worse fit than an unconstrained model, meaning that none of the effects differed significantly between tracks, χ^2^(8) = 6.259, *p* = 0.618.

#### Achievement

Initial levels of school-track-related stereotype awareness in Grade 5 were significantly and positively related to initial levels of achievement in the lowest track *(b* = 0.647, *p* < 0.001), whereas the relationships were not statistically significant in the two other tracks (RQ3, see Table 2). Initial levels of stereotypes were further significantly and negatively related to developmental trajectories in achievement, when initial levels of achievement were controlled for, in the combined track *(b* = −0.314, *p* = 0.046; RQ4). Developmental trajectories in stereotype awareness were not significantly related to developmental trajectories in achievement in any of the tracks (RQ5). Results from model comparisons indicated that the constrained model had a significantly worse fit than the unconstrained one, χ^2^(8) = 50.983, *p* < 0.001, revealing significant differences between tracks. Follow-up tests with the Benjamini-Hochberg correction indicated that for RQ3, the effect in the lowest track differed significantly from effects in the higher track and the combined track (both *p*s < 0.001). The combined and higher tracks did not differ significantly (*p* = 0.128). For RQ4, no differences between tracks were found (lowest track vs. higher track: *p* = 0.738; lowest track vs. combined track: *p* = 0.738; combined track vs. higher track: *p* = 0.884). For RQ5, follow-up tests showed significant differences between the higher track and the combined track (*p* = 0.038). The effect in the lowest track did not differ significantly from the effects in the higher track (*p* = 0.673) or the combined track (*p* = 0.244).

#### Self-concept of academic aptitude

Initial levels of school-track-related stereotype awareness in Grade 5 were significantly and negatively related to initial self-concept levels in the higher track *(b* = −0.251, *p* = 0.001; RQ3). None of the relationships between initial stereotype awareness and changes in self-concept were statistically significant. Changes in stereotype awareness were not significantly related to changes in self-concept. There were no statistically significant differences in the effects between tracks, χ^2^(8) = 12.427, *p* = 0.133.

## Discussion

Secondary school tracking generates inequalities in opportunities and has pervasive consequences for individuals’ educational trajectories and life paths^32^. Despite the widespread use of tracking in Germany and other countries and efforts to identify psychological barriers that further widen school-track-related inequalities in education^11^, stereotypes have received surprisingly little attention to date^3,7,9,33^. The present 4-year longitudinal study on students’ awareness of negative school-track-related stereotypes therefore advances current knowledge in important ways.

We found significant mean-level differences in school-track-related stereotype awareness between all three tracks for all waves in the expected direction, with students from the lowest track consistently reporting higher levels of stereotype awareness than those from the higher and combined tracks. These findings present an important replication and extension of prior work^7^, documenting significant differences between students from the lowest track (Hauptschule, also included in our data) and students from Gymnasium, the highest ability track in Germany. Our finding also add to differentiation-polarization theory^9,10,12,15^ by suggesting that the polarization component can be applied to students’ awareness of negative school-track-related stereotypes, with the most negative perceptions in the lowest track.

Further, negative school-track-related stereotypes increased in all tracks. From a developmental perspective, secondary school tracking coincides with the onset of adolescence, a period in which students’ cognitive capacities to understand the social implications of their academic placement grow^11^. A potential explanation for these ascending trajectories that could apply to students from all three of the tracks we investigated stresses the fact that school-track-related stereotype awareness referring to one’s own school track likely develops in reference to other tracks. Over time and as the end of compulsory schooling nears, restricted opportunities (e.g., for students’ further education and career) linked to their current track as compared with the next higher track(s) may become more apparent to students. Importantly, this phenomenon should also apply to many students from the relatively highest track in our study, who still tend to have fewer opportunities than students from Gymnasium, the highest academic track in Germany, which was not represented in our data.

Next, we investigated links between stereotype awareness and academic outcomes. To summarize the main findings, we did not find support for any of our hypotheses longitudinally, as changes in students’ stereotype awareness were not related to changes in their academic achievement, self-concept of academic aptitude, or engagement.

It was shown that stereotype awareness in Grade 5 significantly predicted a more maladaptive development of achievement over the course of 4 years in the combined track, but the effects did not differ significantly between tracks. Cross-sectionally, we obtained some limited evidence for the “stereotype awareness as harmful for all” hypothesis but only for student engagement. With respect to self-concept of academic aptitude, we found a significant negative cross-sectional relationship to stereotype awareness for students in the higher track; however, the effects for self-concept were not significantly different across the three tracks. Moreover, a positive relationship between stereotype awareness in Grade 5 and achievement was obtained for the lower track, and this effect differed significantly from the effects in the higher track (a nonsignificant small negative relationship) and combined track (a nonsignificant small positive relationship). The cross-sectional effect for academic achievement in the lower track was in line with the “stereotype awareness as an indicator of missed opportunities” hypothesis. None of the obtained effects confirmed the assumptions outlined in the “stereotype awareness as an amplifier of educational inequalities” hypothesis.

Jointly, our findings are consistent with the view that stereotype awareness goes along with lower levels of some aspects of academic functioning, as indicated by negative cross-sectional relationships with engagement in all tracks and a negative cross-sectional relationship with self-concept of academic aptitude in the higher track. The findings for engagement revealed that students who started secondary school with higher levels of negative stereotype awareness were less engaged and reported enjoying school less. Although the effect for self-concept did not differ across tracks, it suggests that for higher track students, the awareness of negative stereotypes about their track was ingrained into their self-perceptions to a higher degree at the beginning of secondary school. Students in the higher track in our data (“Realschule”) were probably more likely to better understand their position and the implications of their track placement, including the awareness that they were not assigned to the highest ability track (i.e., Gymnasium). This understanding could strengthen the link between stereotype awareness and self-concept found in Grade 5. The explanation resembles the mechanisms outlined in the integration paradox. The integration paradox describes the phenomenon that relatively more highly educated immigrants turn away from the host society instead of becoming more oriented toward it^34^.

The cross-sectional positive relationship with academic achievement for students from the lowest track is in contrast with the negative cross-sectional relationships for engagement and self-concept and casts new light on relationships between school-track-related stereotype awareness and achievement^7,9^. We interpret this effect as providing support for the “stereotype awareness as an indicator of missed opportunities” hypothesis. Specifically, the awareness of negative school-track-related stereotypes may serve as an indicator of missed opportunities such that higher achieving students, for whom the next higher track could also have been an option due to their relatively higher achievement levels, report higher levels of awareness of stereotypes that refer to their current track. A reason for why significant effects were restricted to the lowest track students could be that for relatively high-achieving students from the lowest track, positive and self-worth-protecting comparison processes that students from other tracks could use (“I might not have made it to the next higher track, but at least I am not in the next lower one!”) do not work. Therefore, missed educational opportunities may be perceived as particularly drastic^35^ and could go hand in hand with negative school-track-related stereotypes. A potential explanation for why the same pattern was not obtained for students from the combined track, which represents the relatively lower track in the federal state of Saxony, could be that the combined track offers different educational opportunities and types of leaving exams. Therefore, students from this track may feel less “stuck,” and thus, they are less prone to suffer from the mechanisms outlined in the “stereotype awareness as an indicator of missed opportunities” hypothesis. Of course, at this point, our explanations are purely speculative and should be tested empirically in future work.

Finally, stereotype awareness in Grade 5 significantly predicted a more maladaptive development of achievement over the course of 4 years in the combined track, a finding that may indicate that initial stereotype awareness signals resignation and disappointment with one’s track placement that could feed into poorer performance over time. Nonetheless, the sizes of the effects in the three tracks were very similar, and, despite the differentiated pattern of significant and nonsignificant results, the effects did not differ significantly between tracks. Even though we did not obtain any significant relationships between developmental trajectories in stereotype awareness and developmental trajectories in achievement or any of the other academic outcomes, it is worth mentioning that the negative effect for achievement in the higher track just failed to reach statistical significance and differed significantly from the effect in the combined track.

Our study has implications for theory and the understanding of how school-track-related stereotype awareness operates in real life. In short, it is complex and differs from what would be expected on the basis of related research in the laboratory (e.g., on stereotype threat^8^). These differences are likely due to the multifaceted experiences that students have in complex social environments, dynamic shifts in environments and reference groups, students’ subjective interpretations thereof, along with (variations in) developmental processes during adolescence. Hence, we propose that research and future theory development regarding school-track-related stereotype awareness cannot afford to be blind to “the context,” adolescents’ respective meaning making, and dynamic shifts in their perceptions. Further, a developmental (longitudinal) perspective is crucial for disentangling concurrent and longitudinal associations. Relatedly, a more extensive formulation of a theory of school-track-related stereotype awareness in real-life contexts is also informed by what we did *not* find. Specifically, clarity on the potential implications of stereotype awareness in terms of consistent longitudinal relationships with the investigated outcomes could not be achieved. Nonetheless, given the, to the best of our knowledge, lack of longitudinal research on stereotype awareness spanning several years of adolescents’ school careers, the present study’s findings significantly contribute to the existing body of research. What we now need are context-sensitive developmental studies that can capture stereotype awareness on different time scales. For example, longer-term longitudinal assessments could be combined with intensive longitudinal assessments at critical time points (e.g., the transition to secondary school) to shed light on dynamic changes in stereotype awareness, implications for academic outcomes, and interactions with changes in comparison processes and reference group effects.

Our work has implications for practice and policy too. In the present study, students in the lowest track reported the highest levels of stereotype awareness in all waves. Hence, we see a need to counteract school-track-related stereotypes in daily interactions with students and in the media. Still, efforts to curb stereotypes should not be constrained to the lowest track, as relatively higher tracks may also be negatively affected (e.g., Realschule in our data). Moreover, we caution that it is not only about improving the image of tracks but also about improving the actual life realities and chances for students in these tracks. In Germany, secondary school tracking occurs early, track-related upward mobility is still very limited, and the permeability of the education system likely interacts with family background characteristics^35^. In addition to streaming students at a later point in their educational careers, coaching and other types of interventions^36^—especially prior to and at the transition to secondary school and with a focus on socioeconomically disadvantaged students and their parents—could provide a remedy. Students who missed the next higher track could be another relevant target group for interventions, as in our study, relatively higher achieving students in the lowest track reported higher levels of negative school-track-related stereotype awareness right after starting secondary school.

Several limitations and directions for future research should be noted. First, in our study, stereotype awareness was assessed solely in reference to a student’s own track. Future research could gain insights into social comparison processes by investigating how these stereotype awareness ratings differ from students’ awareness of stereotypes about other school tracks. Relatedly, it would be interesting to explore the extent to which the perception that a much stronger stigma is associated with one’s track than with other tracks (i.e., larger stereotype awareness gaps) drives relationships between stereotypes and outcome variables. Second, the awareness of negative stereotypes was assessed and analyzed as a single, general construct, which ensured comparability of the stereotype construct across tracks. At the same time, however, this measurement approach falls short of capturing the existence of potentially different types of stereotypes for students in different tracks. For example, stereotypes about students in higher tracks may be that they are nerdy, boring, socially awkward, elitist, “know-it-alls” or teacher’s pets^7^. Future research should thus include a larger number of more diverse school-track-related stereotypes. Nonetheless, we believe that the content of the stereotype measure we employed was still suitable for our study. Although in our study, the mean levels of stereotype awareness were highest in the lowest track (Hauptschule), significant relationships between stereotype awareness and the outcome variables were in several instances obtained for (or even restricted to) students from Realschule, the higher track. This latter finding indicates that the negative stereotypes we assessed are relevant to higher track students. Third, substantially distinct stereotypes likely exist for the highest ability track (Gymnasium), but this track was not represented in our data.

Fourth, the present study focused on outcomes that reflect academically relevant features in three different domains (academic achievement: actual performance; self-concept of academic aptitude: motivational self-beliefs; engagement: affective school involvement) and that are arguably proximal to academically relevant stereotypes (e.g., “stupid,” “not interested in school”). On the other hand, this focus necessarily excluded other critical outcomes. Future studies on school-track-related stereotype awareness should expand the scope of the present investigation to explore relationships between school-track-related stereotype awareness and a range of outcomes in the socioemotional (e.g., well-being), social (e.g., social networks), emotional (e.g., academic emotions), and behavioral (e.g., disruptive behavior, school drop-out) domains.

Fifth, several items from the academic self-concept scale measure asked students to evaluate their abilities in comparison with others. However, the measure did not specify who these “others” were. Future studies should enhance clarity by indicating and systematically contrasting different reference groups (e.g., elementary school classmates, new secondary school classmates, friends).

Sixth, lastly, limitations that refer to the composition of the sample can be identified and used to inform future studies. Even though we were able to take advantage of a large and rich data set with four measurement points that spanned students’ lower secondary school careers, it remains a drawback that students from the highest track in Germany (Gymnasium) were not included due to the focus of the TRAIN study on specific school types. In addition, differences in secondary school systems across the German federal states need to be kept in mind when interpreting the findings. Specifically, the comparison between Hauptschule (lowest track) and Realschule (higher track) from the federal state of Baden-Württemberg is unequivocal, whereas caution is warranted when comparing these two tracks with Mittelschule in Saxony.

To conclude, stratified school systems likely create stereotypes about students attending different tracks^7^. Our study showed that students from the lowest track consistently reported higher levels of negative school-track-related stereotype awareness than students attending the higher and combined tracks. However, the findings on links between the awareness of school-track-related stereotypes and academic outcomes did not indicate that school-track-related stereotype awareness amplifies educational inequalities. As an initial longer term longitudinal investigation of school-track-related stereotype awareness exploring patterns of effects in different school tracks, our study notably adds to the literature and contributes to a more differentiated understanding of students’ awareness of the stigma associated with their school track.

## Method

### Sample

We used previously collected data from a large-scale longitudinal German study (TRAIN) with four measurement points, which is hosted by the Hector Research Institute of Education Sciences and Psychology at the University of Tübingen in Germany (https://uni-tuebingen.de/en/43704). The TRAIN study was based on a multistage sampling design, in which school-type-specific subpopulations of interest (from the three tracks Hauptschule, Realschule, and Mittelschule) were drawn disproportionately to the actual population shares. The TRAIN study relied on stratified cluster samples, which were drawn separately for both participating federal states (Baden-Württemberg, Saxony). First, a random sample of schools (cluster) was determined for both federal states. From each school, fifth-grade classes were then randomly selected, and all students in these classes were asked to participate in the TRAIN study. This sampling strategy was employed due to the TRAIN study’s focus on the three school tracks of Hauptschule, Realschule, and Mittelschule and allowed for detailed analyses of students and schools from these three tracks. However, the sample is therefore not representative of the population (Rose et al.^37^). The sampling procedure used by the TRAIN study resulted in a target sample of 22 schools for Mittelschule, 25 schools for Realschule, and 60 schools for Hauptschule, all of which were contacted and invited to participate. Out of the schools that were invited, one school from the Realschule track and one school from the Hauptschule track did not participate. The student response rates for the survey assessments were 83–89%, 90–93%, and 74–80% in the Hauptschule, Realschule, and Mittelschule tracks, respectively^37^.

The sample analyzed in this study contained data from 3880 secondary school students enrolled in 136 classes from two German federal states (Baden-Württemberg, 66%, and Saxony, 34%), for whom data from at least one measurement point was available. Across all measurement points, 45.2% of the students identified as female, and they were, on average, 14.20 years old at the fourth measurement point (*SD* = 0.65). A total of 43% of the students attended the academically least demanding track (lowest track, Hauptschule), 23% attended the higher track (Realschule), and 34% attended the combined track (Mittelschule). The students from Hauptschule and Realschule in our sample were from the German federal state of Baden-Württemberg. Most German states, including Baden-Württemberg, track students in the threefold school system consisting of Gymnasium (the highest track), Realschule (the next higher track), and Hauptschule (the lowest track). Hauptschule, the school track with the lowest academic demands, is mainly vocationally oriented. The Realschule curriculum is focused on general education but also lays the foundation for future vocational careers. In comparison with Hauptschule, Realschule provides students with much better opportunities for acquiring higher educational qualifications. Lastly, Gymnasium represents the academically most demanding track, and successfully completing Gymnasium entitles students to study at university^2,38^. In addition, the sample included students from the German federal state of Saxony attending a combined track, Mittelschule. Students from Mittelschule could acquire a Hauptschule diploma or a Realschule diploma. It should further be noted that, like Baden-Württemberg, the secondary school system in Saxony also includes Gymnasium. However, there are no Realschule or Hauptschule tracks in Saxony. Hence, for our study, comparisons between students from Hauptschule (referred to as the lowest track) and Realschule (referred to as the higher track) from Baden-Württemberg are straightforward; however, any comparisons between these two tracks and Mittelschule (referred to as the combined track) should take into consideration the fact that the students from Mittelschule came from another German federal state (Saxony) with a slightly different secondary school system. All variables used in this study were assessed four times, when students were on average 11 (Grade 5), 12 (Grade 6), 13 (Grade 7), and 14 (Grade 8) years of age. All assessments took place some weeks after the start of the respective school year. The study was approved by the state authorities of Baden-Württemberg and Saxony, who, at this time, were responsible for approving studies like this one. Parental consent was required for study participation.

### Measures

Achievement was measured with standardized tests. Stereotypes, self-concept of academic aptitude, and school engagement were assessed via student reports on a 4-point scale ranging from 1 (*completely disagree*) to 4 (*completely agree*). Students’ migration background was measured with student reports, whereas family socioeconomic status was captured with parent reports. Gender was assessed with student and teacher ratings combined into one variable (e.g., in cases in which students did not report their gender, information from teacher reports was used).

### Awareness of school-track-related stereotypes

We used a measure of negative school-track-related stereotypes referring to a student’s own secondary school track^7^. The items were introduced with the following phrase: “What do you think? How do other people in general think about students attending [your school track]? Other people think that typical students from [your school track] are ….” Students were asked to rate the “typical student” from their school track on nine adjectives referring to negative stereotypes with cognitive (“stupid,” “unimaginative,” “dumb”), motivational (“unmotivated in class,” “not interested in school,” “lazy”), and social content (“rude,” “cheeky,” “brazen”). Reliability coefficients (Cronbach’s alpha) for the awareness of school-track-related stereotypes measure for the four waves were .921, .929, .918, and .942 for students from the lowest track; .916, .896, .914, and .935 for students from the higher track; and .932, .923, .929, and .935 for students from the combined track, respectively.

### Academic achievement

Two indicators of academic achievement were used, namely, standardized achievement test scores in mathematics and German. The tests included standard content from the federal states’ mathematics curricula (e.g., arithmetic rules, linear equations, and angels) and German language curricula (i.e., reading comprehension). Open ended, closed ended, and multiple-choice response formats were used (for more detailed descriptions, see refs. ^39,40^). Item and person parameters for students’ mathematics and German achievement have previously been estimated with longitudinal, multidimensional, two-parameter item response theory models^37^, and we relied on weighted likelihood estimators (WLEs) of students’ mathematics and German achievement test scores^41^, that is, one indicator for each subject and wave. To capture school achievement more broadly, we built an average of students’ mathematics and German test scores for the analyses of this study.

### Self-concept of academic aptitude

Students’ self-concept of academic aptitude was measured with four reverse-coded items (“Frequently, I’m convinced that I won’t be able to solve a task even before I get started”; “I frequently think that I’m not as smart as others are”; “I’d like to be as intelligent as others are”; “Compared with others, I’m not as talented”)^42^. Reliability coefficients (Cronbach’s alpha) for the four waves were .668, .714, .727, and .748 for students from the lowest track; .717, .712, .773, and .776 for students from the higher track; and .735, .779, .772, and .776 for students from the combined track, respectively.

### School engagement

We used three items to map school engagement. The items were based on the BIJU study (“I enjoy working on my tasks at school”; “In the morning, I look forward to a day at school to learn something new”; “School is a place I enjoy being at”)^43^. Reliability coefficients (Cronbach’s alpha) for the four waves were .746, .727, .729, and .740 for students from the lowest track; .775, .824, .769, and .765 for students from the higher track; and .782, .756, .769, and .770 for students from the combined track, respectively.

### Covariates

The control variables gender (0 = *female*, 1 = *male*), migration background (0 = *no migration background*, 1 = *migration background*), and socioeconomic background (SES; highest socio-economic index of occupational status of the parents, HISEI^30^) were considered. Specifically, these control variables were included in all analyses in which effects on academic outcomes were estimated (i.e., the analyses for RQ3–RQ5).

### Measurement models and measurement invariance testing

We tested the factor structure of all multiple-item scales with confirmatory factor analyses (CFAs) conducted in M*plus* Version 8.6^44^, including longitudinal measurement invariance testing and testing for invariance between school tracks. For growth curve models (main analyses), at least scalar invariance (equal item intercepts and factor loadings) is needed^45^. For stereotypes, we furthermore compared models with different numbers of stereotype awareness factors. Originally, scholars distinguished between students’ awareness of cognitive, social, and motivational school-track-related stereotypes^7^, and for our study, we tested for whether these stereotypes represent (a) three distinct facets, (b) one overarching construct, or (c) two constructs by comparing three-factor, two-factor, and one-factor CFA models. We assessed the goodness of fit of all models using the comparative fit index (CFI), the Tucker-Lewis Index (TLI), and the root mean square error of approximation (RMSEA). Typical cut-off scores that are considered to reflect excellent and adequate fit to the data, respectively, were considered: (a) CFI and TLI > .95 and >.90, (b) RMSEA < 0.05 and <0.08^46^.

The evaluation of longitudinal and intergroup invariance assumptions was based on respective recommendations from the methodological literature^45,47,48^. Hence, we considered drops in the CFI or TLI > 0.01 and increases in the RMSEA > 0.015 as indicative of meaningful changes in model fit, which make assumptions of measurement invariance untenable. When relying on latent factors, several problems can occur, and it is not possible to anticipate all of them. For instance, there may be single items that show a substantially low(er) loading on the latent factors than others, and model fits might not be in line with traditional recommendations^46^. When this was the case, we carefully checked both statistical indicators (e.g., lower loading, worse model fit) and content-related indicators (e.g., a specific item might not “represent” the respective construct as well as other items do) and adapted our models to achieve adequate fit. Accordingly, for school engagement, we decided to use the three (positively worded) items from the original seven-item scale that best captured students’ broader affective school engagement based on the respective CFA (and additionally conducted exploratory factor analysis) results and conceptual considerations. Moreover, for the school engagement scale, residuals from the same items were allowed to correlate across time.

For stereotype awareness, the CFA findings showed that a two-factor model with a factor for cognitive stereotype awareness and a factor combining social and motivation stereotype awareness fit the data slightly better than the next best one-factor solution (two-factor model: CFI = .954, TLI = .949, RMSEA = 0.031; one-factor model: CFI = .942, TLI = .938, RMSEA = 0.034). The three-factor model did not converge. In addition, it should be noted that we only included the negatively worded items because including both positively worded (reverse-coded) and negatively worded items resulted in a very poor model fit and convergence problems (introducing a method factor did not solve the problem). Conceptually, as our aim was to measure the awareness of negative stereotypes, focusing on the negatively worded items seems appropriate, and we therefore excluded the three positively worded statements for cognitive, social, and motivational stereotype awareness, respectively (e.g., “smart,” “polite”). There was a deviation from our preregistered analysis plan for the main analyses (see the “Statistical analyses” section for the main analyses). On the basis of the CFA results, we had preregistered that we would model cognitive and social/motivational stereotype awareness separately. However, despite the slightly superior fit of the respective two-factor solution in comparison with a one-factor solution, a closer inspection revealed that the two stereotype awareness factors were strongly correlated. Whereas the manifest correlations ranged from .78 to .83 (which was slightly lower than those previously reported^7^, the latent correlations ranged from .90 to .97. We therefore decided to rely on one overall stereotype awareness factor in our (latent) main analyses. Tables reporting detailed CFA and measurement invariance testing results for all constructs can be found in the Supplementary Tables 2–4. To summarize, both longitudinal invariance and invariance between tracks could generally be established, and the main analyses (see Statistical analyses) were based on the respective models.

### Statistical analyses

All analyses were performed with M*plus* Version 8.6^44^ using the robust maximum likelihood estimator (MLR), which is robust to non-normal data. To deal with missing data, we employed full information maximum likelihood estimation (FIML^49^). All multiple-item scales were modeled as latent variables. Achievement was modeled as a manifest indicator for which mathematics and German test scores were combined.

For RQ1, we tested for mean level differences in stereotype awareness between the three tracks separately for each wave. Specifically, we tested for mean differences in latent stereotype awareness factors by constraining them to equality between groups and then comparing the constrained model with a model with unconstrained stereotypes. This comparison was done jointly, and then we compared individual groups as a follow-up test (including the adjustment of *p*-values with the Benjamini-Hochberg correction).

Next, we set up multigroup latent growth curve models. To investigate developmental trajectories in school-track-related stereotype awareness (RQ2), we estimated univariate growth models. To allow greater flexibility for the shape of the curve, we estimated latent basis growth models in which only the first and the last growth parameters were fixed, whereas all other parameters were freely estimated. Thereby, no linearity was imposed on the models. However, the growth parameters were set to be equal between the groups (tracks) so that we could meaningfully compare the slopes between them.

For RQ3–RQ5, we estimated multigroup growth curve models to examine relationships between initial levels (intercepts) of stereotype awareness and initial levels (intercepts) of all outcomes (RQ3), relationships between initial levels (intercepts) of stereotype awareness and changes (slopes) in all outcomes (RQ4), as well as relationships between changes (slopes) in school-track-related stereotype awareness and changes (slopes) in all outcome variables (RQ5). We created separate models for each outcome due to model complexity. All models included time-invariant covariates (SES, gender, migration background). We evaluated differences in parameters between the groups by using a nested-models χ^2^ approach with follow-up comparisons to isolate the source of the overall differences. To account for the hierarchical data structure, with students nested in classes, the analyses were conducted with cluster-robust standard errors. All significance testing was performed at the 0.05 level, and we relied on two-tailed tests. Benjamini-Hochberg corrections^50^ were used to adjust for multiple tests. We applied the adjustment for each research question separately (but across the three different outcomes) and adjusted all *p*-values that were relevant for our research questions^51,52^.

### Supplementary information

Supplementary Figures and Tables

## Data Availability

The data are currently not publicly available due to data privacy/ethical restrictions. Data used for this submission may be made available upon request to collaborators of the TRAIN study research team (https://uni-tuebingen.de/en/faculties/faculty-of-economics-and-social-sciences/subjects/department-of-social-sciences/education-sciences-and-psychology/research/aktuelle-studien/train/).

## Code availability

All analysis code is available on the OSF (anonymized link: https://osf.io/uwjrm/?view_only=ef06f871bcb34447929c8ac34fe277f1).
