# Supplementary material for: Does students’ awareness of school-track-related stereotypes exacerbate inequalities in education?
Source: NPJ Sci Learn. 2023 Dec 14;8:59. doi: 10.1038/s41539-023-00203-9 (PMC10721808; doi:10.1038/s41539-023-00203-9)
Supplement: Supplementary file 1 — Supplementary Figures and Tables [file 41539_2023_203_MOESM1_ESM.pdf]

Supplementary Figures

Supplementary Figure 1

Bivariate (Latent) Correlations Between All Variables for the Higher Track

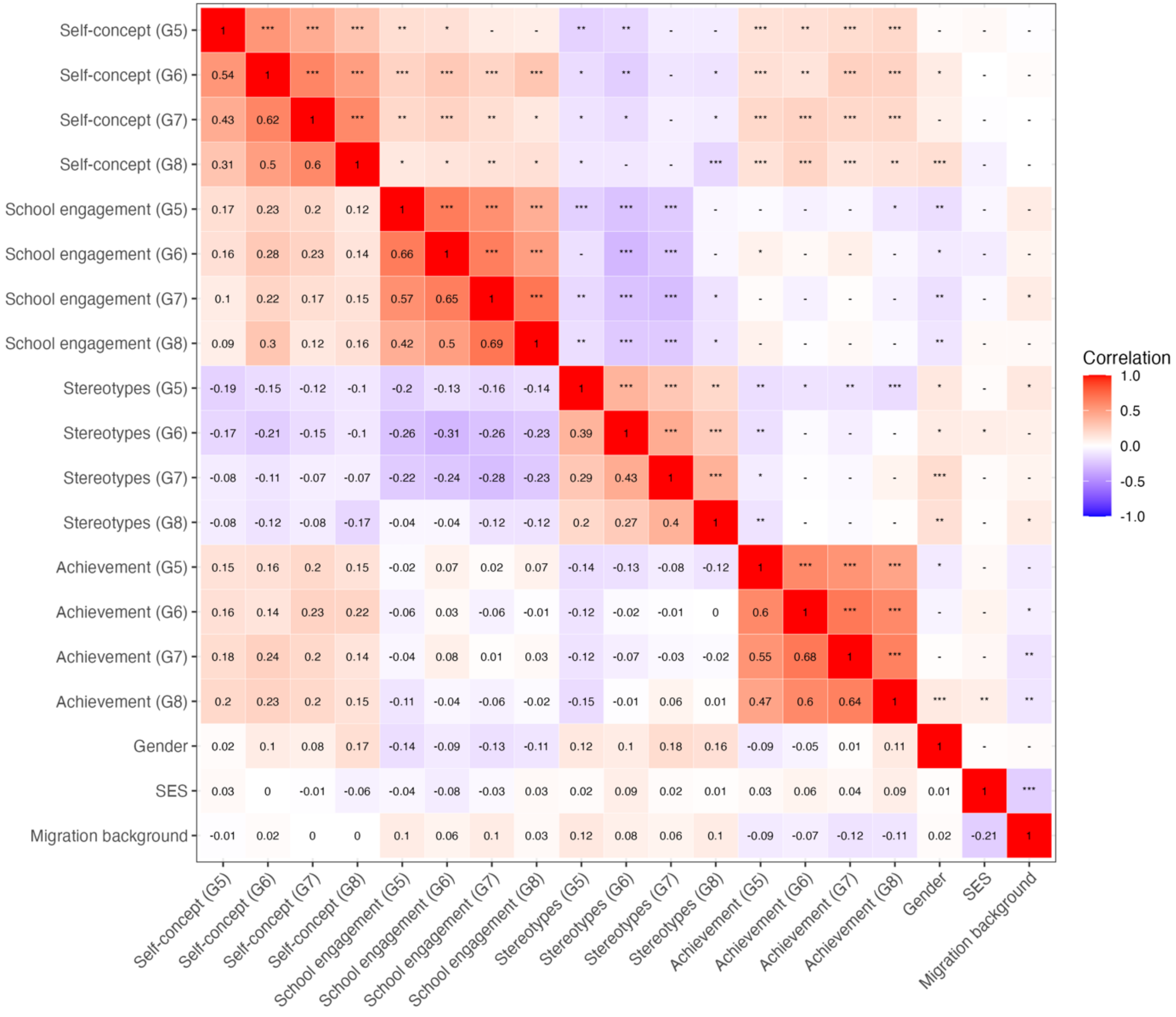

Note. Correlation coefficients are presented below the diagonal, and information on statistical significance (*$p < .05$. **$p < .01$. ***$p < .001$) is presented above the diagonal. G5–G8 = Grades 5–8.

Supplementary Figure 2

Bivariate (Latent) Correlations Between All Variables for the Combined Track

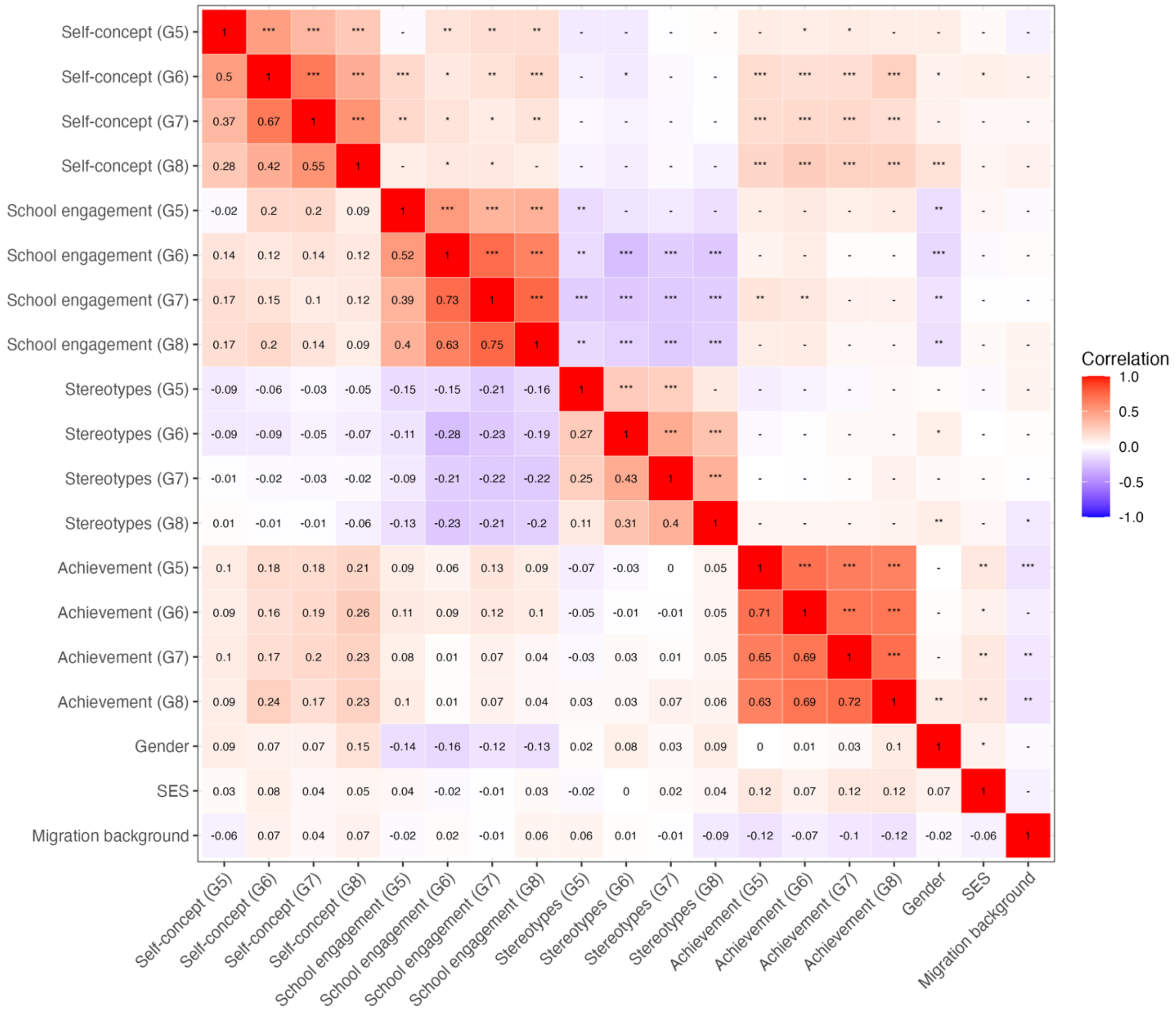

Note. Correlation coefficients are presented below the diagonal, and information on statistical significance (**p* < .05. ***p* < .01. ****p* < .001) is presented above the diagonal. G5–G8 = Grades 5–8.

Supplementary Figure 3

Bivariate (Latent) Correlations Between All Variables for the Lowest Track

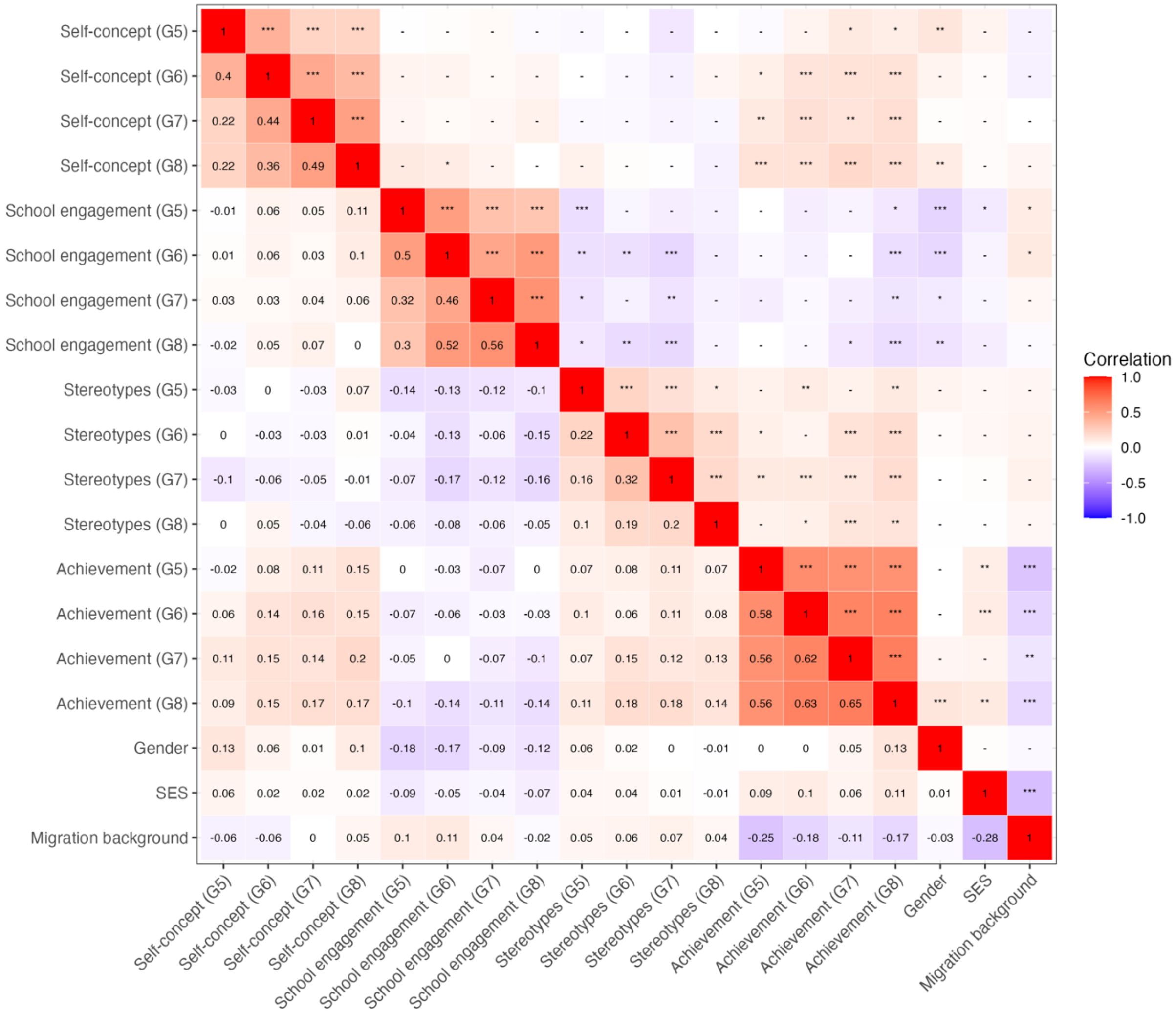

Note. Correlation coefficients are presented below the diagonal, and information on statistical significance (**p* < .05. ***p* < .01. ****p* < .001) is presented above the diagonal. G5–G8 = Grades 5–8.

Supplementary Tables

Supplementary Table 1

Descriptive Statistics for All Variables

**Variable**	**Lowest track**		**Combined track**		**Higher track**		**Overall**
	M	*SD*	*M*	*SD*	*M*	*SD*	*Missing*
Gender	0.56	--	0.55	--	0.53	--	0.3%
SES	43.21	11.86	45.54	12.11	49.46	13.44	15.2%
Migration background	0.47	--	0.05	--	0.17	--	40.0%
Self-concept Grade 5	2.7	0.79	2.93	0.75	2.95	0.71	45.6%
Self-concept Grade 6	2.75	0.77	2.77	0.74	2.86	0.71	39.7%
Self-concept Grade 7	2.77	0.77	2.78	0.72	2.91	0.71	37.2%
Self-concept Grade 8	2.74	0.75	2.78	0.68	2.81	0.71	35.9%
School engagement Grade 5	2.9	0.86	2.52	0.85	2.77	0.78	45.3%
School engagement Grade 6	2.64	0.84	2.34	0.76	2.58	0.8	39.2%
School engagement Grade 7	2.57	0.8	2.26	0.74	2.46	0.73	36.7%
School engagement Grade 8	2.46	0.76	2.17	0.7	2.34	0.71	35.7%
Stereotypes Grade 5	2.27	0.92	2.12	0.8	1.83	0.74	46.1%
Stereotypes Grade 6	2.36	0.91	2.21	0.75	1.87	0.65	39.1%
Stereotypes Grade 7	2.49	0.87	2.31	0.76	1.96	0.69	37.2%
Stereotypes Grade 8	2.55	0.93	2.41	0.76	2.14	0.77	39.4%
Achievement Grade 5	0.04	0.76	0.75	0.86	1.17	0.75	30.0%
Achievement Grade 6	0.33	0.86	1.11	0.95	1.47	0.9	33.5%
Achievement Grade 7	0.65	0.89	1.45	1.01	1.83	0.9	29.0%
Achievement Grade 8	0.98	1.08	2.02	1.26	2.18	1.14	29.6%

Note. *N* (lower track) = 853–1,674; *N* (combined track) = 606–1,315; *N* (highest track) = 556–827. Gender: 0 = female, 1 = male. Migration background: 0 = without, 1 = with; SES was measured with the HISEI (highest socio-economic index of occupational status in the family, Ganzeboom et al., 1992). Descriptive statistics are based on manifest variables. *M* = Mean, *SD* = Standard deviation.

Supplementary Table 2

Preliminary Factor Analysis for Stereotype Awareness: Model Comparisons

Models	CFI	TLI	RMSEA
T1			
1 overall factor	.948	.930	0.074
2 factors (social and cognitive vs. motivational)[1]	.948	.928	0.076
2 factors (social and motivational vs. cognitive)	.949	.929	0.075
T2			
1 overall factor	.956	.941	0.07
2 factors (social and cognitive vs. motivational)[1]	.957	.940	0.071
2 factors (social and motivational vs. cognitive)	.965	.951	0.064
T3			
1 overall factor	.944	.926	0.076
2 factors (social and cognitive vs. motivational)[a]	.945	.924	0.077
2 factors (social and motivational vs. cognitive)	.960	.944	0.066
T4			
1 overall factor	.957	.942	0.068
2 factors (social and cognitive vs. motivational)[1]	.956	.939	0.069
2 factors (social and motivational vs. cognitive)	.964	.95	0.063
T1–T4 in one model			
1 overall factor	.942	.938	0.034
2 factors (social and cognitive vs. motivational)[1]	.943	.937	0.034
2 factors (social and motivational vs. cognitive)	.954	.949	0.031

Note. Stereotype models containing one or two factors were compared. Three-factor models could not be estimated due to convergence problems (collinearity of factors).

[a] Factors are perfectly correlated.

Supplementary Table 3

Confirmatory Factor Analysis and Fit Statistics for Latent Factors

Models	CFI	TLI	RMSEA
**Self-concept of academic aptitude**			
Self-concept (congeneric factor model)*	.950	.939	0.034
Self-concept (tau-equivalent factor model)	.868	.856	0.052
Self-concept (tau-equivalent factor model with autocorrelations)	.914	.880	0.048
Self-concept (parallel factor model)	.790	.798	0.062
Self-concept (parallel factor model with autocorrelations)	.832	.800	0.061
**School engagement**			
School engagement (parallel factor model)	.921	.923	0.050
School engagement (parallel factor model with autocorrelations)*	.960	.946	0.042
**Stereotype Awareness**			
Stereotypes (congeneric factor model)	.942	.938	0.034
Stereotypes (tau-equivalent factor model)*	.935	.934	0.035
Stereotypes (parallel factor model)	.919	.922	0.038
Stereotypes (parallel factor model with errors fixed across time)	.916	.920	0.039
Stereotypes (parallel factor model with errors fixed across time and autocorrelations)	.925	.922	0.038

Note. Confirmatory factor analyses were conducted across the whole sample to explore the fit for more parsimonious measurement models. Parallel factor models contain items with similar loadings and error variances. Tau-equivalent factor models contain similar loadings but unconstrained error variances. Congeneric factors can contain different loadings and different error variances. Autocorrelations of the same items across time were permitted for some models to improve fit and account for item-specific method variance.

*These factor models were used in the analyses.

Supplementary Table 4

Measurement Invariance Testing Results: Longitudinal Invariance and Invariance Across Groups (Tracks)

Models	CFI	TLI	RMSEA
**Self-concept of academic aptitude**			
Within tracks			
Lowest track			
No longitudinal invariance	0.941	0.927	0.033
Weak longitudinal invariance	0.941	0.933	0.032
Strong longitudinal invariance	0.933	0.931	0.033
Combined track			
No longitudinal invariance	0.957	0.947	0.036
Weak longitudinal invariance	0.956	0.951	0.034
Strong longitudinal invariance	0.955	0.954	0.034
Higher track			
No longitudinal invariance	0.949	0.937	0.038
Weak longitudinal invariance	0.948	0.942	0.037
Strong longitudinal invariance	0.944	0.942	0.037
Between tracks			
No between-group invariance	0.945	0.943	0.034
Weak between-group invariance	0.945	0.944	0.034
Strong between-group invariance	0.943	0.943	0.034
**School engagement**			
Within tracks			
Lowest track			
Weak longitudinal invariance	0.970	0.960	0.031
Strong longitudinal invariance	0.957	0.948	0.036
Combined track			
Weak longitudinal invariance	0.925	0.899	0.062
Strong longitudinal invariance	0.917	0.901	0.061
Higher track			
Weak longitudinal invariance	0.965	0.953	0.045
Strong longitudinal invariance	0.952	0.943	0.050
Between tracks			
Weak between-group invariance	0.919	0.921	0.052
Strong between-group invariance	0.91	0.914	0.054
**Stereotypes**			
Within tracks			
Lowest track			
Weak longitudinal invariance	0.934	0.933	0.035
Strong longitudinal invariance	0.932	0.933	0.035
Combined track			
Weak longitudinal invariance	0.922	0.921	0.040
Strong longitudinal invariance	0.919	0.921	0.040
Higher track			
Weak longitudinal invariance	0.902	0.901	0.044
Strong longitudinal invariance	0.897	0.899	0.044
Between tracks			
Weak between-group invariance	0.918	0.92	0.039
Strong between-group invariance	0.916	0.919	0.039

Note. First, strong temporal invariance was tested within each group. Second, invariance across time and group was tested. Strong invariance was achieved by constraining the loadings and intercepts of the items to be equal across time and group. For school engagement (measurement invariance testing within tracks: lowest track and higher track), changes in the CFI for the strong invariance models in comparison with the weak invariance

models were slightly above the cut-off of .01 (i.e., .013), but changes in the RMSEA were in favor of strong invariance.

Supplementary Table 5

Multigroup Linear Basis Growth Curve Model Parameters for the Developmental Trajectory of Stereotype Awareness in the Three School Tracks

School Track	**Lowest Track**				**Combined Track**				**Higher Track**			
Parameter	*b*	*SE*	*Est/SE*	*p*	*b*	*SE*	*Est/SE*	*p*	*b*	*SE*	*Est/SE*	*p*
**Mean structure**												
Intercept of stereotypes	2.099	0.036	58.965	<.001	1.942	0.033	59.524	<.001	1.603	0.031	52.251	<.001
Slope of stereotypes	0.281	0.04	6.975	<.001	0.288	0.045	6.393	<.001	0.329	0.043	7.696	<.001
**Covariance structure**												
Intercept of stereotypes	0.22	0.035	6.199	<.001	0.21	0.031	6.701	<.001	0.202	0.026	7.886	<.001
Slope of stereotypes	0.229	0.12	1.907	.057	0.311	0.129	2.403	0.016	0.246	0.109	2.262	.024
Intercept-slope-covariance	-0.109	0.061	-1.793	.073	-0.103	0.056	-1.842	0.066	-0.09	0.049	-1.859	.063
**Growth parameters**												
Grade 5	0				0				0			
Grade 6	0.265	0.071	3.758	<.001	0.265	0.071	3.758	<.001	0.265	0.071	3.758	<.001
Grade 7	0.567	0.073	7.814	<.001	0.567	0.073	7.814	<.001	0.567	0.073	7.814	<.001
Grade 8	1				1				1			

Note. A linear basis model was estimated, that is, the growth parameters of the first and the last time point were set to 0 and 1, respectively, whereas other growth parameters were estimated freely to allow for nonlinear growth trajectories. Growth parameters were set equal across the three groups. Unstandardized parameters are presented.
